# Assessment of General and Cardiac Toxicities of Astemizole in Male Cynomolgus Monkeys: Serum Biochemistry and Action Potential Duration

**DOI:** 10.5487/TR.2008.24.4.289

**Published:** 2008-12-01

**Authors:** Jong-Hwa Lee, Do-Geun Kim, Joung-wook Seo, Hyang-Ae Lee, Jeong-Hwa Oh, Ho-Chul Shin, Seok Joo Yoon, Choong-Yong Kim

**Affiliations:** 17 Research and Development Division, Korea Institute of Toxicology, 100 Jang-dong, Yuseong, Daejeon, 305-343 Korea; 27 Division of Non-clinical Studies, Korea Institute of Toxicology, Daejeon, 305-343 Korea; 37 Department of Veterinary Pharmacology and Toxicology, College of Veterinary Medicine, Konkuk University, Seoul, 143-701 Korea

**Keywords:** Astemizole, Cardiotoxicity, Cynomolgus monkey, APD, Serum biochemistry

## Abstract

Toxicology screening following treatment with astemizole, a histamine receptor antagonist, at oral doses of 0, 10, 30 and 60 mg/kg was carried out in male cynomolgus monkeys (*Macaca fascicularis*). No dose-related changes in mortality, clinical signs, body weight changes, food consumption, or urine analysis occurred in any animal compared to the vehicle control. However, the high-dose group showed a decrease in BUN and ALP compared to vehicle control group. In addition, the levels of TG, AST, ALP and CK increased. Although astemizole did not produce significant toxicological changes at any dose tested, we predict that it can cause toxicological changes of the liver and heart based on the changes in the serum parameters related to the heart and liver. The Action Potential Duration (APD) was prolonged in the heart of 60 mg/kg treatment group compared to the control group. The APD increase in 60 mg/kg treatment group along the other related changes in toxicological parameters imply that astemizole has major cardiotoxic effects in the cynomolgus monkey. This study is a valuable assessment for predicting the general toxicity and cardiotoxic effects of antihistamine drugs using nonhuman primates.

## INTRODUCTION

Astemizole, a second-generation $H_1$-antihistamine drug that was introduced in the mid-1980s, was used widely for 10 years to alleviate the symptoms of allergy and urticaria. However, the use of terfenadin and astemizole was limited after studies revealed that they could induce QT prolongation and ventricular tachyarrhythmia or torsade de pointes, which can be fatal (Rao *et al.*, 1994; Paakkari, 2002). Astemizole induce cardiotoxicity by blocking Ikr (the delayed rectifier potassium channel), which causes ventricular tachyarrhythmia (Vorperian *et al.*, 1996).

Although the toxicity of antihistamine had to be determined revealed at the early stage of drug development, because of the genetic differences between humans and laboratory animals such as rats and mice, the efforts were not sufficiently thorough. To overcome this shortcoming, using non-human primates is necessary in the toxicity screening of antihistamines.

Haemtological and blood serum chemistryare frequently used to screen the toxicity of drugs as basic data for interpreting the result of preclinical studies (Matsuzawa *et al.*, 1993; Kim *et al.*, 2004).

The action potential duration (APD) is used to assess the conductance of heart muscle in animal models of conductive heart disease including atrioventricular block (Nouchi *et al.*, 2008). Recently, this test was used to confirm the effect of channel blockers in delaying conductivity (Milberg *et al.*, 2008).

In this study, we evaluated the changes in mortality, clinical signs, body weight, urinalysis, haematology, serum biochemistry, and APD test in cynomolgus monkey *(Macaca fascicularis)* after the oral administration of astemizole as antihistamine to elucidate the changes in toxicological parameters implied that it has major cardiotoxic effects in cynomolgus monkey.

## MATERIALS AND METHOD

***Animals.*** Male cynomolgus monkeys (*Macaca fascicularis*) were from China and acclimated in Non-Human Primate Animal Room in Korea Institute of Toxicology prior to use. They were examined for any abnormalities suggestive of health problems. At initiation of dosing, the age range was from 4~7 years and the body weight range was 4493~6266 g.

***Husbandry.*** The animal room was maintained at a temperature 23 ± 3°C, relative humidity 55 ± 10%, luminous intensity of 150~300 Lux, ventilation rate of 10~20 times/hour, and air pressure of negative and over 3 mmAq. A 12-hour light/dark cycle (lighting 07:00~19:00) was used. The temperature and relative humidity of animal room was monitored daily and recorded continuously. According to the monitoring report, no significant deviations affecting the experiment were observed.

Primate diet (PS, Orient Yeast Co., Japan) was offered daily to 4% of the weight of the animals during the study period. A certificate of analysis for the diet was supplied by provider and KIT. The result of analysis indicated no effect on the study. UV-sterilzed and filtered municipal tap water was given *ad libitum.* No significant contamination that would be expected to affect the experimental results was detected in the water.

***Dosage and group assessment.*** There were 4 groups (3 animals/group) dosing 0, 10, 30 and 60 mg/kg/day for the toxicity study. Based on the result that astemizole 30 mg/kg/day induced QT prolongation in nonhuman primates, 30 mg/kg was selected as middle doses and 10 and 60 mg/kg/day were selected to low dose and high dose, respectively. 0.5% MC (MethylCelluose) was used as the vehicle.

***Administration route.*** The oral route of administration was used based on the route of clinical administration. The animals were fasted at least 2 hours before astemizole administration, and the amount of given was based on the most recent weight of the animals.

***Animal care and use.*** All experimental procedures involving animals in this study were reviewed by the Institutional Animal Care and Use Committee (IACUC) of the Korea Institute of Toxicology, according to National Institutes of Health guidelines (NIH publication number 85-23, revised 1985) "*Principles of Laboratory Animal Care*". All animals used in this study were cared for in accordance with the principles outlined in the NIH publication of "*Guide for the Care and Use of Laboratory Animals*".

***Clinical observation and body weight measurement.*** Monkeys were observed once daily for mortality and signs of pain/distress; unscheduled observations were recorded. Abnormal findings were recorded individually for all observations. The body weight of each monkey was recorded using a balance on Day 1 prior to dosing and weekly thereafter.

***Food consumption.*** Food consumption were determined and calculated as mean intake amount for individual (g/monkey/day) for 24 hours. Mean food intake was recorded weekly during the treatment.

***Urinalysis.*** Urine Stick (Multistix 10 SG, Bayer) and automatic tester (CliniTek-500, Bayer) were used to analyze specific gravity, pH, glucose, bilirubin, protein, ketone body, occult blood, nitrite and urobilinogen. Urine sedimentation test was performed to examine red blood cell, white blood cell and columnar epithelial cell under light microscope. $Na^+$, $K^+$ and $Cl^-$ were automatically measured by the automatic biochemical analyzer (TBA 200FR Neo, Toshiba, Japan).

***Haematology.*** Approximately 0.5 ml of blood was collected in EDTA-2K tubes (CBC, Sewon medical Co.) via cephalic, saphenous or femoral vein on 48 hour after treatment. Automatic blood cell counter (ADVIA 120 Haematology system, Bayer, USA) was used to perform haematological analysis. The items evaluated are shown in Table 1. Reticulocyte was counted using light microscope after staining with methylene blue. The percentage of white blood cell was obtained by counting 100 cells in a blood smear slide stained with Wright-Giemsa using light microscope and calculated the percentage of neutrophil, eosinophil, basophil, lymphocyte and monocyte.

***Serum biochemistry.*** Blood samples (approximately 1 ml) were collected without an anticoagulant via cephalic vein. The collected samples were placed at room temperature for at least 90 minutes and centrifuged at 1500 g for 10 minutes to obtain serum. Serum were analyzed for alanine transaminase (ALT), aspartate trasaminase (AST), alkaline phosphatase (ALP), triglyceride (TG), total cholesterol (TCHO), glucose (GLU), total protein (TP), albumin (ALB), blood urea, nitrogen

**Table 1.** Abbreviation, unit and analysis methods of the items

| Items | Unit | Methods |
|---|---|---|
| WBC (White blood cell count) | × $10^3/mm^3$ | Laser Optical with cytochemical reaction |
| RBC (Red blood cell count) | × $10^6/mm^3$ | Laser Optical (Flow cytometry) |
| HGB (Hemoglobin concentration) : Hb | g/dl | Cyanmethemoglobin Spectrophotometry |
| HCT (Hematocrit) : Ht | % | Calculation From MCV |
| MCV (Mean corpuscular volume) | fl | Laser Optical (Flow cytometry) |
| MCH (Mean corpuscular hemoglobin) | pg | (HGB/RBC) × 10 |
| MCHC (Mean corpuscular hemoglobin concentration) | g/dl | (HGB/(RBCXMCV)) × 1000 |
| PLT (Platelet) | × $10^3/mm^3$ | Laser Optical (Flow cytometry) |
| Reticulocyte count | % | Laser Optical with cytochemical reaction |
| Differential leucocyte count | % | Perox optical with chemical reaction |
| AST (Aspartate aminotransferase) | IU/l | UV-Rate |
| ALT (Alanine aminotransferase) | IU/l | UV-Rate |
| ALP (Alkaline phosphatase) | IU/l | P-NPP |
| BUN (Blood urea nitrogen) | mg/dl | UV rate |
| CREA (Creatinine) | mg/dl | Jaffe |
| GLU (Glucose) | mg/dl | Hexokinase |
| TCHO (Total cholesterol) | mg/dl | Enzyme |
| A/G (Albumin globulin ratio) | ratio | ALB/(TP-ALB) |
| TP (Total protein) | g/dl | Biuret |
| ALB (Albumin) | g/dl | BCG |
| CK (Creatine kinase) | IU/l | UV-Rate |
| TG (Triglyceride) | mg/dl | Enzyme |
| TBIL (Total bilirubin) | mg/dl | Enzyme |
| PL (Phospholipid) | mg/dl | Enzyme |
| IP (Inorganic phosphorous) | mg/dl | UV |
| $Ca^{2+}$ (Calcium) | mg/dl | OCPC |
| $Cl^-$ (Chloride) | mmol/l | Electrode |
| $Na^+$ (Sodium) | mmol/l | Electrode |
| $K^+$ (Potassium) | mmol/l | Electrode |

(BUN), creatine kinase (CK), creatinine (CREA), phospholipid (PL), total bilirubin (TBIL), gamma-glutamyl transpeptidase (GGT), inorganic phosphorous (IP), lactate dehydrogenase (LDH), albumin/globulin ratio (A/G), calcium ($Ca^{2+}$) using automatic blood serum chemistry analyzer (Shimadzu CL-7200, Shimadzu Co., Japan), and sodium ($Na^+$), potassium ($K^+$) and chloride ($Cl^-$) was analyzed by automatic electrolyte analyzer (644, $Na^+$/ $K^+$/ $Cl^-$ analyzer Ciba-corning, USA).

***Action potential duration (APD) test.*** To confirm if astemizole administered by oral route can prolong the action potential of heart muscle by suppressing blocking Ikr channel, Action Potential Duration test which evaluates the QT prolongation in heart muscle. This study was conducted in facilities approved by the AAALAC (Association for Assessment and Accreditation of Laboratory Animal Care) International. All procedures were approved by our Institutional Animal Care and Use Committee (IACUC). Purkinje fibers were isolated from the left ventricles of hearts from vehicle control (n = 3) and 60 mg/kg/day (n = 1), sacrificed by overdose with sodium pentobarbitone. Spontaneously beating Purkinje fibers were mounted in a continuous flow (5 ml/min) and temperature controlled (37 ± 1°C) chamber superfused with normal Tyrode (NT) solution (in mM): 145 NaCl, 5.4 KCl, 5 HEPES, 0.33 $NaH_2PO_4$, 0.5 $MgCl_2$, 16.6 glucose, 1.8 $CaCl_2$. The NT solution was oxygenated with $O_2$ gas. Throughout the experiment, the preparation was stimulated via silver bipolar electrodes at suprathreshold levels (frequency = 1 Hz, duration = 2 ms, voltage = 1.5~2 V) to evoke cardiac action potentials. Action potentials were recorded using a standard glass microelectrode filled with 3 M KCl (resistance at 10~30 M). Action potentials were amplified using Geneclamp 500B (Molecular Devices Corp., CA, USA), and data were stored and analyzed using the NotocordHEM program (NOTOCORD, France). Action potential duration at 50% ($APD_{50}$) and 90% ($APD_{90}$) of repolarization, total amplitude (TA), resting membrane potential (RMP) and Vmax of the phase 0 max depolarization values were determined.

***Statistical analysis.*** Numerical data obtained during the conduct of the study were subjected to calculation of group means and standard deviations and were reported in the final report. The data were analyzed for homogeneity of variance using Bartlett's test. Homogeneous data were analyzed using the Analysis of Variance and the significance of inter-group differences were analyzed using Dunnett's t test. Heterogeneous data were analyzed using Kruskal-Wallis test and the significance of inter-group differences between the control and treated groups were assessed using Dunn's Rank Sum test. Statistical analyses were performed by comparing the different dose groups with the vehicle control group using Path/Tox System and Statistical Analysis Systems (SAS/STAT Version 8.1, Cary, USA).

## RESULT AND DISCUSSION

In this study, we administered astemizole to cynomolgus monkeys (*Macaca fascicularis*) in a single oral dose of 10, 30 and 60 mg/kg/day to assess its toxicity. The data obtained were compared to those of a vehicle control group.

No differences in mortality, clinical signs, body weight changes, urinalysis, or food consumption compared were observed compared to the control group (Table 2~4). The mucous stools from Day 1 to Day 16 and soft stools from Day 6 to Day 16 found in some animals were determined to not a toxic effect of astemizole.

No significant changes were observed in haematological analysis, although the numbers of neutrophils and lymphocytes were elevated on Day 2 compared to the vehicle control (Table 5). The percentage of lymphocytes has been reported to decrease with increasing age, while the percentage of neutrophil increases (Wolford *et al.*, 1987).

**Table 2.** Mortality and clinical signs in cynomologus monkeys after a single dose of astemizole

| | Dosing Group | | | |
|---|---|---|---|---|
| | V.C (0 mg/kg) | T1 (10 mg/kg) | T2 (30 mg/kg) | T3 (60 mg/kg) |
| **Final mortality** | | | | |
| Day 1 | 0/3[a)] | 0/3 | 0/3 | 0/3 |
| ≤ Day 5 | 0/3 | 0/3 | 0/3 | 0/3 |
| ≤ Day 10 | 0/3 | 0/3 | 0/3 | 0/3 |
| ≤ Day 20 | 0/3 | 0/3 | 0/3 | 0/3 |
| **Clinical signs** | | | | |
| Day 1~Day 20 | -[b)] | - | - | 1/3 (Soft stool)<br>1/3 (Mucous stool)<br>1/3 (No stool) |

[a)]Values were expressed as number of dead or clinical signing animals/total number of animals.
[b)]No clinical signs.

**Table 3.** Body weight and food consumption in cynomolgus monkeys after a single dose of astemizole

| | Dosing group | | | |
|---|---|---|---|---|
| | V.C (0 mg/kg) | T1 (10 mg/kg) | T2 (30 mg/kg) | T3 (60 mg/kg) |
| **Mean body weight** | | | | |
| Day 1 | 6266.3 ± 1235.6 | 4392.7 ± 56.8 | 4504.3 ± 738.7 | 6234.7 ± 870.9 |
| Day 8 | 6226.7 ± 1194.2 | 4427.3 ± 36.1 | 4569.7 ± 693.4 | 6194.7 ± 839.8 |
| Day 15 | 6236.7 ± 1193.7 | 4481.7 ± 78.8 | 4662.7 ± 600.2 | 6197.7 ± 817.6 |
| **Food consumption** | | | | |
| Day 1 | 120.2 ± 0.2 | 120.7 ± 0.2 | 119.8 ± 0.3 | 120.4 ± 0.3 |
| Day 8 | 120.1 ± 0.1 | 120.1 ± 0.1 | 120.3 ± 0.1 | 120.1 ± 0.1 |
| Day 15 | 120.2 ± 0.2 | 120.1 ± 0.1 | 120.1 ± 0.1 | 120.1 ± 0.1 |

Each value represents mean ± S.D. (n = 3).

**Table 4.** Urinalysis of cynomolgus monkeys orally treated with astemizole

| | Dosing Group | | | |
|---|---|---|---|---|
| | V.C (0 mg/kg) | T1 (10 mg/kg) | T2 (30 mg/kg) | T3 (60 mg/kg) |
| U-K | 36.5 ± 28.8 | 28.7 ± 8.5 | 31.3 ± 16.6 | 30.8 ± 19.7 |
| U-CL | 17.0 ± 5.1 | 17.0 ± 7.8 | 15.0 ± 3.1 | 14.0 ± 6.1 |
| U-Na | 18.0 ± 8.4 | 18.0 ± 8.4 | 10.0 ± 3.2 | 10.0 ± 2.6 |

**Table 5.** Haematological values in cynomolgus monkeys after a single dose of astemizole

| | Day 2 | | | | Day 14 | | | |
|---|---|---|---|---|---|---|---|---|
| | 0 mg/kg | 10 mg/kg | 30 mg/kg | 60 mg/kg | 0 mg/kg | 10 mg/kg | 30 mg/kg | 60 mg/kg |
| WBC (K/ul) | 9.5 ± 0.7 | 10.8 ± 2.0 | 12.9 ± 3.1 | 9.1 ± 4.4 | 9.5 ± 1.5 | 10.5 ± 1.4 | 11.7 ± 1.8 | 9.7 ± 3.5 |
| RBC (M/ul) | 5.6 ± 0.2 | 5.4 ± 0.5 | 5.3 ± 0.2 | 5.6 ± 0.2 | 5.6 ± 0.4 | 5.4 ± 0.4 | 5.4 ± 0.2 | 5.8 ± 0.3 |
| HGB (g/dl) | 13.0 ± 0.4 | 13.2 ± 0.5 | 12.7 ± 0.9 | 13.4 ± 0.5 | 13.5 ± 0.5 | 13.8 ± 0.1 | 13.3 ± 1.0 | 14.1 ± 0.4 |
| HCT (%) | 42.9 ± 0.6 | 42.2 ± 0.4 | 42.9 ± 3.5 | 42.8 ± 1.0 | 42.8 ± 2.0 | 42.5 ± 1.5 | 43.5 ± 2.2 | 43.9 ± 1.1 |
| MCV (fl) | 76.6 ± 3.2 | 79.0 ± 7.7 | 80.7 ± 8.1 | 76.6 ± 1.3 | 76.6 ± 3.3 | 78.5 ± 9.0 | 81.1 ± 6.4 | 75.9 ± 1.6 |
| MCH (pg) | 23.3 ± 1.5 | 24.7 ± 2.1 | 23.8 ± 2.3 | 24.0 ± 0.6 | 24.2 ± 2.0 | 25.5 ± 1.8 | 24.9 ± 2.5 | 24.4 ± 0.5 |
| MCHC (g/dl) | 30.4 ± 0.8 | 31.4 ± 0.9 | 29.6 ± 0.6 | 31.3 ± 0.8 | 31.6 ± 1.3 | 32.5 ± 1.3 | 30.6 ± 0.8 | 32.1 ± 0.1 |
| PLT (K/μl) | 413.0 ± 35.0 | 480.0 ± 55.7 | 481.0 ± 120.7 | 456.0 ± 92.9 | 438.0 ± 33.0 | 436.0 ± 87.6 | 496.0 ± 125.5 | 448.0 ± 83.6 |
| NEU (%) | 39.4 ± 7.0 | 29.7 ± 13.9 | 52.1 ± 16.2 | 57.9 ± 5.7 | 40.5 ± 13.6 | 24.7 ± 7.5 | 36.1 ± 9.0 | 59.2 ± 15.4 |
| LYM (%) | 55.6 ± 4.7 | 66.0 ± 13.4 | 41.4 ± 13.1 | 31.7 ± 11.0 | 53.8 ± 11.8 | 70.2 ± 7.3 | 55.5 ± 4.7 | 33.0 ± 10.0 |
| MONO (%) | 3.0 ± 1.0 | 2.2 ± 0.4 | 4.7 ± 2.5 | 3.9 ± 2.2 | 2.7 ± 0.9 | 2.6 ± 0.6 | 4.3 ± 2.3 | 3.2 ± 1.0 |
| ESO (%) | 1.1 ± 1.1 | 1.4 ± 1.1 | 0.8 ± 0.3 | 5.3 ± 6.2 | 1.3 ± 1.0 | 1.4 ± 1.1 | 2.2 ± 1.5 | 3.5 ± 4.6 |
| BASO (%) | 0.3 ± 0.1 | 0.2 ± 0.1 | 0.2 ± 0.2 | 0.2 ± 0.2 | 1.1 ± 0.9 | 0.5 ± 0.3 | 1.3 ± 0.8 | 0.5 ± 0.3 |
| LUC (%) | 0.6 ± 0.4 | 0.5 ± 0.3 | 0.7 ± 0.3 | 1.0 ± 1.1 | 0.6 ± 0.2 | 0.6 ± 0.2 | 0.8 ± 0.1 | 0.5 ± 0.1 |

Each value represents mean ± S.D. (n = 3).

**Table 6.** Serum biochemical values in cynomolgus monkeys after a single dose of astemizole

| | Day 2 | | | | Day 14 | | | |
|---|---|---|---|---|---|---|---|---|
| | 0 mg/kg | 10 mg/kg | 30 mg/kg | 60 mg/kg | 0 mg/kg | 10 mg/kg | 30 mg/kg | 60 mg/kg |
| AST (IU/l) | 54.2 ± 22.6 | 53.7 ± 14.9 | 44.0 ± 8.8 | 102.3 ± 113.4 | 40.7 ± 13.8 | 45.3 ± 16.3 | 38.4 ± 8.0 | 41.5 ± 4.3 |
| ALT (IU/l) | 82.7 ± 52.3 | 59.3 ± 12.1 | 87.0 ± 21.1 | 100.7 ± 27.7 | 64.6 ± 38.7 | 43.2 ± 20.0 | 33.3 ± 2.9 | 43.0 ± 5.0 |
| ALP (IU/l) | 622.3 ± 248.9 | 1002.3 ± 520.1 | 619.6 ± 283.9 | 482.9 ± 29.8 | 652.4 ± 176.7 | 1008.0 ± 502.2 | 663.0 ± 350.4 | 543.9 ± 57.8 |
| BUN (mg/dl) | 17.2 ± 2.0 | 17.9 ± 1.6 | 20.5 ± 2.8 | 14.4 ± 4.4 | 18.7 ± 1.2 | 19.6 ± 2.0 | 22.3 ± 1.0 | 12.5 ± 2.5** |
| CREA (mg/dl) | 1.1 ± 0.1 | 1.0 ± 0.0 | 1.1 ± 0.1 | 1.0 ± 0.1 | 1.2 ± 0.1 | 1.0 ± 0.1 | 1.2 ± 0.1 | 1.1 ± 0.2 |
| GLU (mg/dl) | 54.9 ± 4.6 | 62.6 ± 6.5 | 63.0 ± 8.9 | 55.6 ± 10.7 | 53.9 ± 5.4 | 53.1 ± 7.4 | 62.2 ± 13.3 | 68.3 ± 19.9 |
| TCHO (mg/dl) | 109.7 ± 5.9 | 115.0 ± 21.3 | 122.7 ± 16.3 | 117.7 ± 14.5 | 116.0 ± 18.0 | 110.7 ± 22.1 | 133.3 ± 27.4 | 125.3 ± 2.5 |
| A/G (ratio) | 1.2 ± 0.1 | 1.2 ± 0.1 | 1.1 ± 0.1 | 1.0 ± 0.0 | 1.2 ± 0.1 | 1.2 ± 0.1 | 1.2 ± 0.1 | 1.1 ± 0.1 |
| TP (g/dl) | 23.8 ± 0.5 | 32.8 ± 5.6 | 41.1 ± 6.7 | 39.4 ± 9.0 | 7.6 ± 0.3 | 6.9 ± 0.8 | 7.1 ± 0.3 | 7.7 ± 0.8 |
| ALB (g/dl) | 4.2 ± 0.1 | 3.9 ± 0.2 | 3.8 ± 0.0 | 4.0 ± 0.2 | 4.1 ± 0.1 | 3.7 ± 0.4 | 3.8 ± 0.1 | 3.9 ± 0.3 |
| CK (IU/l) | 156.0 ± 56.5 | 156.0 ± 43.6 | 252.0 ± 142.5 | 1718.0 ± 2686.7 | 122.0 ± 43.7 | 174.0 ± 57.0 | 194.0 ± 90.9 | 167.0 ± 86.2 |
| TG (mg/dl) | 23.8 ± 0.5 | 32.8 ± 5.6 | 41.1 ± 6.7* | 39.4 ± 9.0* | 24.7 ± 3.7 | 32.2 ± 3.6 | 43.3 ± 12.9 | 46.3 ± 9.7* |
| TBIL (mg/dl) | 0.2 ± 0.0 | 0.2 ± 0.0 | 0.2 ± 0.0 | 0.2 ± 0.1 | 0.2 ± 0.0 | 0.2 ± 0.0 | 0.1 ± 0.0 | 0.1 ± 0.0 |
| PL (mg/dl) | 165.0 ± 13.9 | 187.0 ± 45.2 | 188.0 ± 41.2 | 187.0 ± 9.2 | 162.0 ± 22.7 | 170.0 ± 32.9 | 173.0 ± 50.2 | 173.0 ± 29.9 |
| IP (mg/dl) | 5.0 ± 1.4 | 5.7 ± 0.2 | 5.5 ± 1.6 | 4.9 ± 0.5 | 4.8 ± 0.9 | 5.3 ± 0.5 | 5.7 ± 1.2 | 4.7 ± 0.8 |
| $Ca^{2+}$ (mg/dl) | 9.5 ± 0.2 | 9.3 ± 0.3 | 9.6 ± 0.3 | 9.7 ± 0.4 | 10.0 ± 0.2 | 9.5 ± 0.4 | 10.1 ± 0.3 | 10.0 ± 0.5 |
| $Cl^-$ (mmol/l) | 109.0 ± 1.7 | 109.0 ± 3.6 | 110.0 ± 4.7 | 110.0 ± 1.7 | 108.0 ± 0.6 | 108.0 ± 1.2 | 109.0 ± 3.1 | 106.0 ± 2.1 |
| $Na^+$ (mmol/l) | 151.0 ± 1.0 | 149.0 ± 3.2 | 153.0 ± 7.6 | 151.0 ± 2.3 | 148.0 ± 0.6 | 147.0 ± 2.5 | 151.0 ± 3.1 | 147.0 ± 2.5 |
| $K^+$ (mmol/l) | 5.0 ± 0.2 | 4.8 ± 0.3 | 5.0 ± 1.2 | 4.9 ± 0.2 | 4.6 ± 0.4 | 4.2 ± 0.6 | 4.7 ± 0.7 | 4.1 ± 0.1 |

Each value represents mean ± S.D. (n = 3).
The statistical significant difference is indicated (*p < 0.05, **p < 0.01).

In serum biochemistry, A significant increase ($P < 0.05$) in TG in the 30 and 60 mg/kg/day monkeys and decrease ($P < 0.01$) in BUN in the 60 mg/kg/day monkeys were observed (Table 6). In addition, AST, ALT and CK tended to increase, while ALP tended to decrease. The decreased in BUN was caused by either hepatocellular dysfunction or a problem with the hepatocellular conversion of ammonia to urea (Walter *et al.*, 1989). As the TG concentration in blood can be affected by factors such as the amount of lipid consumed, intestinal absorption, TG synthesis and excretion in the liver, and TG absorption and storage in lipid tissue, the cause of the increase in TG is uncertain.

The AST, ALT and CK increased (Table 6) above the physiological range (AST, 31.7~76.5 IU/l; ALT, 30.4~87.7 IU/l; CK, 216~791 IU/l; Kim *et al.*, 2004). Of these enzymes, AST has high activity in heart muscle, and is used as a marker of heart toxicity. In addition, AST exists in the liver, kidney, and brain. With liver disease, the AST and ALT levels increase. Therefore, the levels of these enzymes evaluate liver function. ALT is excreted mainly from the liver; it shows high activity in the cell membrane without necrosis and is thus is used as a biomarker for hepatocellular injury. CK shows high

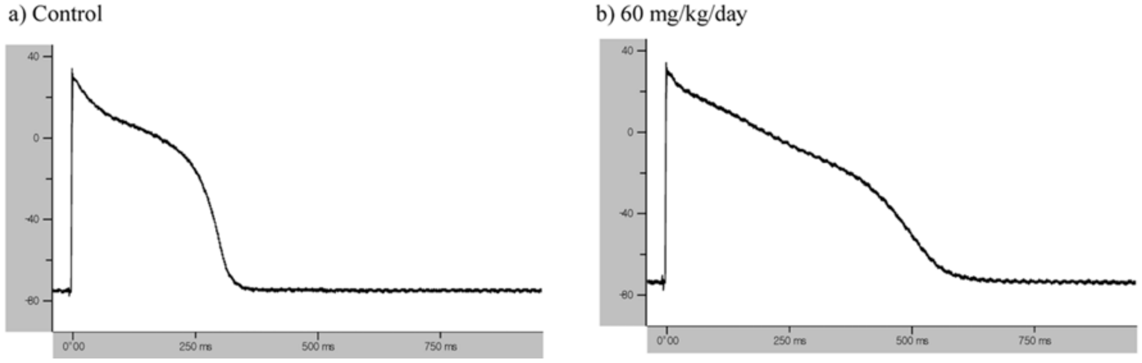

**Fig. 1.** Representation of ventricular action potential duration changes in control (n = 3) and treatment group (n = 1).

**Table 7.** Changes in action potential duration

| Parameter | 0 mg/kg (n = 3) | | 60 mg/kg (n = 1) |
|---|---|---|---|
| | Mean | SEM | |
| RMP (mV) | -77.3 | 1.1 | -70.6 |
| TA (mV) | 115.2 | 1.3 | 97.5 |
| $APD_{50}$ (ms) | 281.2 | 25.7 | 339.4 |
| $APD_{90}$ (ms) | 382.0 | 22.1 | 600.3 |
| Vmax (V/s) | 351.7 | 55.6 | 267.1 |

*Abbreviation: RMP: resting membrane potential, TA: total amplitude, Vmax: maximal upstroke velocity of phase 0, $APD_{50}$: action potential duration at 50%, $APD_{90}$: action potential duration at 90%.

activity mainly in heart, skeletal muscle, and brain and its levels increase markedly in myocardial infarction. Since marked variation was detected within group of 60 mg/kg/day, the increase levels likely resulted not from the astemizole, but from muscle damage caused by restraint.

Although astemizole did not produce significant toxicological changes at any dose tested, we predict that it can cause toxicological changes of the liver and heart based on the changes in the serum parameters related to the heart and liver.

The APD showed QT prolongation in the astemizole-treated animals. In the 60 mg/kg/day dose, $APD_{50}$ and $APD_{90}$ were 339.4 and 600.3 ms, respectively and these increased by 20.7% and 57.1%, respectively, compared to the control group (Fig. 1, Table 7). This was thought to arise from the inhibitory effect of astemizole on Ikr. Finally, the APD increase in group of 60 mg/kg/day compared to the control group along the other related changes in toxicological parameters imply that astemizole has major cardiotoxic effects in the cynomolgus monkey.

We hope that this study provides useful data for predicting the general and cardiotoxic effects of antihistamine drugs using nonhuman primates.

## ACKNOWLEDGEMENTS

This study was conducted with the support of the Ministry of Science and Technology (KK-0803, Developing Leading Safety Assessment Technology using Non-Human Primates, 2008) and we especially thank Phil-Soo Lee, Gu-Hwa Kang, Nam-Su Park and Kang-Hyun Han for contributing greatly to this study.
